# Supplementary material for: Meldonium Inhibits Cell Motility and Wound-Healing in Trabecular Meshwork Cells and Scleral Fibroblasts: Possible Applications in Glaucoma
Source: Pharmaceuticals (Basel). 2023 Apr 15;16(4):594. doi: 10.3390/ph16040594 (PMC10143983; doi:10.3390/ph16040594)
Supplement: Supplementary file 1 [file pharmaceuticals-16-00594-s001.zip › pharmaceuticals-2311312-supplementary.pdf]

Supporting Information

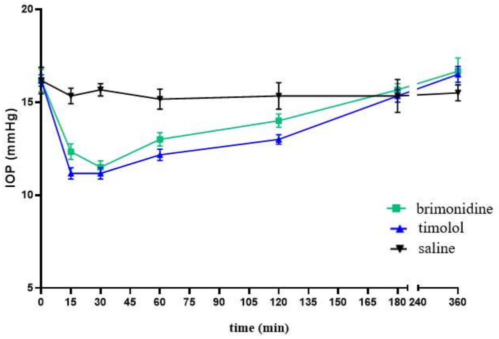

Figure S1. Effect of timolol and brimonidine eye drops on baseline IOP in rats. In each rat (n = 3: 6 eyes) eye drops (10 µl) were instilled in each eye. SE bars are shown. *** p<0.001 (15′-30′) ** p<0.01 (60′) vs saline control.

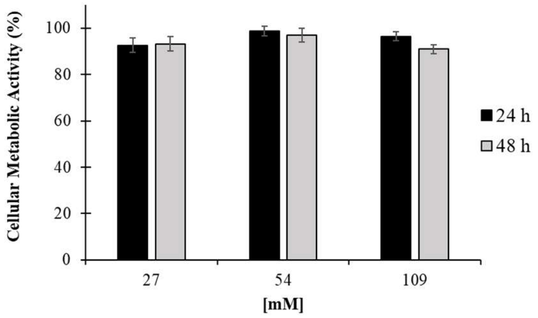

Figure S2. Cell viability (MTT assay) of HSF treated with increasing concentrations of MID for 24 and 48 h in complete culture medium (2% FCS). Data are expressed as means ± S.D. of three independent experiments, each performed in triplicate.
